# Reduced viscosity Barley β-Glucan versus placebo: a randomized controlled trial of the effects on insulin sensitivity for individuals at risk for diabetes mellitus

**DOI:** 10.1186/1743-7075-8-58

**Published:** 2011-08-16

**Authors:** Harold Bays, Joy L Frestedt, Margie Bell, Carolyn Williams, Lore Kolberg, Wade Schmelzer, James W Anderson

**Affiliations:** 1 Louisville Metabolic & Atherosclerosis Research Center, 3288 Illinois Avenue, Louisville, 40213, USA; 2 Frestedt Incorporated, 2708 Vernon Avenue South, Minneapolis, 55416. USA; 3 Biometrics, ClinData Services, Incorporated, 3100 John Hinkle Place, Suite 105, Bloomington, 47408, USA; 4 Louisville Metabolic & Atherosclerosis Research Center, 3288 Illinois Avenue, Louisville, 40213, USA; 5 Scientific and Regulatory Affairs, Cargill Incorporated, 15407 McGinty Rd W, Mailstop #163, Wayzata, 55391, USA; 6 Health and Nutrition, Cargill Incorporated, 15407 McGinty Rd W, Mailstop #163, Wayzata, 55391, USA; 7 Medicine and Clinical Nutrition, University of Kentucky, 506 Knapp Farm Drive, Hermitage, 37076, USA

**Keywords:** insulin sensitivity, insulinemia, glycemia, barley beta glucan, randomized controlled trial

## Abstract

**Background:**

Prior studies suggest soluble fibers may favorably affect glucose/insulin metabolism.

**Methods:**

This prospective, randomized, placebo controlled, double blind, parallel group trial evaluated 50 generally healthy subjects without prior diagnosis of diabetes mellitus (44 completers), who were administered beverages containing placebo (control), lower dose (3 g/d), or higher dose (6 g/d) reduced viscosity barley β-glucan (BBG) extract. Subjects (68% women) mean age 56 years, Body Mass Index (BMI) 32 kg/m^2 ^and baseline fasting plasma glucose 102 mg/dl were instructed to follow a weight-maintaining Therapeutic Lifestyle Changes (TLC) diet and consumed three 11 oz study beverages daily with meals for 12 weeks. The four primary study endpoint measures were plasma glucose and insulin [each fasting and post-Oral Glucose Tolerance Testing (OGTT)].

**Results:**

Compared to placebo, administration of 3 g/d BBG over 12 weeks significantly reduced glucose incremental Area Under the Curve (iAUC) measures during OGTT and 6 g/d BBG over 12 weeks significantly reduced fasting insulin as well as the related homeostasis model assessment of insulin resistance (HOMA-IR). Beverages were generally well tolerated with no serious adverse experiences and no significant differences between groups for adverse experiences. Per protocol instruction, subjects maintained body weight.

**Conclusions:**

These findings suggest 6 g/d BBG consumed in a beverage over 12 weeks may improve insulin sensitivity among hyperglycemic individuals with no prior diagnosis of diabetes mellitus, and who experience no change in body weight.

**Trial Registration:**

ClinicalTrials.gov Identifier: NCT01375803.

## Introduction

Type 2 diabetes mellitus (T2DM) is increasing as a worldwide, major health epidemic [1,2]. Individuals at increased risk for developing T2DM include those who are 45 years old or greater, have a family history of T2DM, lead a sedentary lifestyle, or have high blood pressure. When combined with other risk factors such as serum triglycerides greater than 150 mg/dl or a body mass index (BMI) greater than 30 kg/m^2^, fasting plasma glucose between 91-99 mg/dl may increase the risk of T2DM [3,4]. One of the potential opportunities in assessing early hyperglycemia is that those with recent onset, mild hyperglycemia may be especially responsive to nutritional and lifestyle interventions, with greater improvements in fasting glucose levels [5] and slowed progression towards development of T2DM [6].

Data suggest whole grain consumption or cereal fiber intake may reduce glucose levels, and reduce the risk for developing T2DM [7,8]. A meta-analysis of 13 prospective cohort studies including 427,935 individuals indicated individuals in the highest quintile for whole grain or cereal fiber intake had a 29% lower risk for developing T2DM than those in the lowest quintile [9]. Viscous soluble fiber intake has favorable effects on postprandial glycemia and insulinemia with improved insulin sensitivity, suggesting a potential mechanism for reduced risk for developing T2DM [10-15]. Several prior reports [16-20] suggest BBG, a soluble fiber, may lower postprandial glucose and insulin; however, most of these were single meal test studies of limited duration (i.e. one test meal time point for food consumption).

The purpose of this 12-week study was to evaluate a flavored water beverage containing reduced viscosity BBG on glucose metabolism and insulin sensitivity among generally healthy individuals at risk for T2DM while following a weight neutral diet.

## Materials and methods

### Study Ethics

The protocol, protocol amendments, informed consent documents, applicable privacy regulation authorizations, recruitment materials and written information provided to the subjects were reviewed and approved by Schulman Associates Institutional Review Board (Cincinnati, OH) before any subject participated in any clinical trial activities. Subjects underwent the informed consent process prior to study procedures being performed, as documented by their signature on an informed consent document.

### Study Design

This was a randomized, double blind, placebo controlled, parallel group interventional study.

### Study Subjects

Inclusion criteria were: generally healthy men and women, age 30-70 years with a Body Mass Index (BMI) of 25-40 kg/m^2 ^and a fasting plasma glucose value of 95-140 mg/dl. Subjects were excluded if they were previously diagnosed as having T2DM, food allergy or sensitivity to study product ingredients, celiac disease, uncontrolled hypertension, fasting serum triglyceride >250 mg/dl, untreated hypothyroidism, recent history of weight loss, were pregnant, breast feeding or at risk of becoming pregnant, current or recent history of drug, alcohol or chemical abuse, or if they had used medications or herbal remedies for weight loss within 3 months before treatment in this clinical trial.

### Study Diets and Test Products

After undergoing the informed consent process, study participants underwent a 2 week lead-in period where they were given nutrition instruction to follow a weight-maintaining Therapeutic Lifestyle Changes (TLC) diet regimen throughout the trial [21,22] and received a test dose of 2 consecutive days of placebo raspberry flavored water (to determine study participant willingness and ability to tolerate 3 times a day test beverage consumption). Subjects were interviewed, and instructed to complete a 3-day diary between study visits in order to assess compliance with the weight maintaining TLC nutritional recommendations.

If study participants remained eligible and willing to proceed after the lead-in period, they were randomized in a double-blind fashion to consume placebo (0 g), lower dose (1 g) or higher dose (2 g) reduced viscosity BBG extract (Barliv™ barley betafiber, Cargill, Wayzata, MN) three times daily with meals or food (total daily dose of BBG: 0 g/d, 3 g/d or 6 g/d) over a 12-week period. BBG, a soluble fiber, is Generally Regarded as Safe (GRAS).

Compliance with study product consumption was evaluated by subject interview, by reviewing the study product consumption record which subjects were asked to complete each day and by counting the used and unused study product containers which were returned to the research site at scheduled visits. Protocol non-compliance was defined as consumption of <75% or >125% of the scheduled intakes of the study beverage.

Raspberry flavored beverages with a pH of 3.2 were thermally processed and aseptically packaged into 11 fluid ounce packages. All beverages were lightly sweetened using a combination of 2% sucrose and sucralose, yielding 6.6 grams of sugar per serving of each treatment beverage. Incremental addition of BBG extract to achieve the low and high doses of BBG resulted in 35 and 38 calories per serving, respectively, compared to 32 calories for the placebo (Table 1). BBG content of the beverages was verified after production by Covance Laboratories (Madison, WI) using the American Association of Analytical Chemists method 995.16. No change in BBG content was observed with subsequent analysis after completion of the study.

**Table 1:** Nutrient Content of Flavored Beverages per Individual Serving (11 fl oz)

Amount per Serving	Placebo	Low Dose (1 g)	High Dose (2 g)
**Calories (kcal)**	32	35	38
**Total Fat (g)**	0	0	0
**Total Carbohydrates (g)**	7.3	8.7	10.0
**Sugars (g)**	6.6	6.6	6.6
**B-Glucan (g)**	0	1	2
**Protein (g)**	0	0	0

### Study Visits and Measurements

Six study visits occurred over 14 weeks and fasting blood work was drawn at visits 1, 2, 4, 5 and 6 on weeks -2, 0, 6, 9 and 12. Study visit procedures included a physical exam during visits 2 (randomization) and 6 (end-of-study) which incorporated vital signs and a pregnancy test. Subjects were counseled in the weight-maintaining TLC diet regimen and encouraged to maintain their baseline weight for the 12-week duration of the study. Subjects completed 3-day diet diaries in order to assess compliance with the background diet. BMI was calculated by dividing weight in kilograms (kg) by height in meters (m) squared and reported as kg/m^2^. Fasting blood was drawn for chemistry, plasma glucose and insulin, and homeostasis model assessment of insulin resistance (HOMA-IR) values [23] and Matsuda Index [24,25]. HOMA-IR is a measure of insulin "resistance" (mainly in the liver) and the Matsuda Index is a measure of insulin "sensitivity" (mainly in the muscle). A 75 g glucose drink was administered for the OGTT with subsequent blood draws for measurement of glucose and insulin at 30, 60, 90 and 120 minutes (+/- 5 minutes) after the time of dosing at the second (randomization) and last visits only. A central laboratory (Quest Laboratory, Lexington, KY) was used for laboratory testing and safety was evaluated by reviewing all treatment-emergent adverse events (AE) and any abnormal laboratory values.

### Statistical Methods

The primary endpoint measures were fasting and post-Oral Glucose Tolerance Testing (OGTT) plasma glucose and insulin. Measures at week 0 (baseline/randomization), week 12 (end of study) and the change from week 0 to week 12 were compared between the three groups (0, 3 and 6 g/d BBG). The assumptions of normality were tested using the Shaipro-Wilks test as well as Box-Cox and logarithmic transformation of the outcome variable. If no suitable transformation was found, non-parametric models were used by ranking (r) the measures first before running the statistical model. Characteristics were compared between treatment groups and differences in responses between treatment groups were analyzed by analysis of variance (ANOVA) or Fisher exact tests as appropriate. The ANOVA statistical models were used to assess the impact of the terms for treatment, site and treatment by site interactions which were reduced in a stepwise manner until only significant (p ≤ 0.05) terms or treatment group effects remained. Pair-wise comparisons utilizing Dunnett's procedure were made in order to identify if the active groups differed from the control group. The paired t-test was utilized for within treatment group comparisons. An incremental Area Under the Curve (iAUC) analysis analyzed the timed OGTT data and to compare the values between groups for the change in iAUC for glucose and insulin from baseline to end of study after 12 weeks of treatment. Adverse events were tabulated by first occurrence for each coded term and by body system. Possible differences in the occurrence of adverse events were assessed by Chi-square tests, and if statistically significant (p ≤ 0.05), then Fisher's exact test (two-tailed) was utilized for pair-wise comparisons between each active group and control. Comparisons between treatment groups for changes in serum chemistry, hematology and urinalysis from week 0 to week 12 were assessed by ANOVA with treatment group as factor.

## Results

### Subject Population

**Figure 1:**
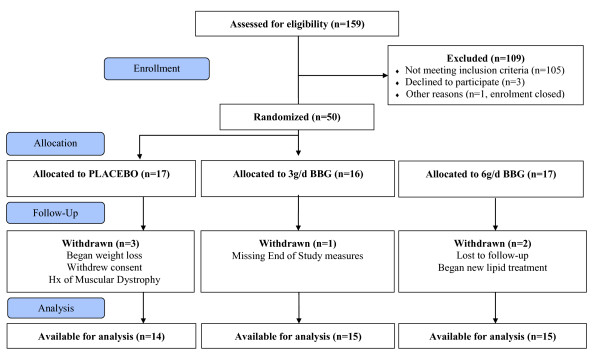
**Subject Disposition Chart**.

One hundred fifty nine (159) subjects meeting eligibility criteria were consented, enrolled and screened; 50 were randomized (placebo = 17; 3 g/d BBG = 16; 6 g/d BBG = 17), with 44 subjects completing the 12 week study (Figure 1). Baseline demographics were comparable among study groups (Table 2). Study participants were mostly women (68%) with an average age of 56 years, BMI 32 kg/m^2^, and predominantly white (88%). Twelve percent were African American. Study subjects were classified according to the American Diabetes Association categories for fasting glucose and 2 hr post OGTT at baseline (Table 3) [3]. Study compliance defined as compliance with beverage consumption for the 12 week study included 97.6%, 99.4% and 97.4% of subjects for the 3 g/d, 6 g/d and placebo respectively. Three subjects in the placebo group and two subjects in the 6 g/d BBG group discontinued the study prior to a post-baseline collection of glucose and insulin; therefore, 14 subjects in the placebo and 15 subjects in the 6 g/d BBG group were analyzed at Week 12. In addition, one subject in the 3 g/d BBG group was missing the week 12 insulin measure; therefore, 15 subjects in the 3 g/d BBG group were analyzed at Week 12.

**Table 2:** Baseline Demographics^1,2^

	Placebo	3 g/d BBG	6 g/d BBG
Parameter	N = 17	N = 16	N = 17
**Female Sex**	10 (58.8%)^3^	11 (68.8%)	13 (76.5%)
**White Race**	15 (88.2%)	14 (87.5%)	15 (88.2%)
**Age (years)**	55.6 (5.2)	55.9 (7.8)	57.1 (6.0)
**Body Weight (kg)**	93.5 (14.1)	90.9 (12.5)	90.1 (14.1)
**Height (cm)**	167.9 (10.5)	169.3 (9.4)	168.4 (11.1)
**BMI (kg/m^2^)**	33.2 (4.1)	31.8 (4.1)	31.8 (4.6)
**Waist Circumference (cm)**	109.2 (9.8)	105.4 (11.3)	105.2 (11.2)
**Systolic Blood Pressure (mm Hg)**	123.9 (12.6)	123.3 (13.8)	120.9 (10.3)
**Diastolic Blood Pressure (mm Hg)**	77.8 (5.2)	78.6 (8.3)	77.4 (6.5)
**Heart Rate (bpm)**	71.2 (9.0)	64.8 (7.4)	68.1 (7.9)

^1 ^P-values for continuous variables were generated by analysis of variance with treatment as factor; P-values for categorical variables were derived by the chi-square test; none were significant (less than 0.05); kcal = kilocalories; kg = kilogram; cm = centimeter; m = meter, mmHg = millimeters of mercury; bpm = beats per minute

^2 ^Data represented includes all randomized subjects

^3^Sex and Race values are presented in this table as N (%) while all other values are presented as Mean (Standard Deviation)

**Table 3:** American Diabetes Association (ADA) Classification at Baseline^1^

Parameter	ADA Classification	Placebo (N = 17)	3 g/d BBG (N = 16)	6 g/d BBG (N = 17)	p-value^2^
**Fasting Plasma Glucose (mg/dL)**	**Normal: Fasting Plasma Glucose < = 99 mg/dL**	9; 95.1 (1.3)	6; 91.7 (2.9)	7; 91.0 (2.5)	0.335
	**Impaired: Fasting Plasma Glucose 100- 125 mg/dL**	8; 110.3 (2.4)	9; 108.2 (2.1)	10; 105.6 (1.0)	0.218
	**Diabetes Mellitus: Fasting Plasma Glucose >= 126 mg/dL**		1; 145.0		
**OGTT (mg/dL): 2 hrs post dose**	**Normal: Glucose at 2 hours < = 139 mg/dL**	9; 94.6 (7.7)	11; 101.3 (8.0)	13; 103.5 (6.8)	0.699
	**Impaired: Glucose at 2 hours 140-199 mg/dL**	5; 169.6 (8.1)	3; 176.3 (13.9)	3; 160.3 (3.7)	0.568
	**Diabetes Mellitus: Glucose at 2 hours >= 200 mg/dL**	3; 212.0 (5.0)	2; 229.5 (2.5)	1; 201.0	0.093

Values presented are N; Mean (Standard Error of Mean)

^1 ^Data represented includes all randomized subjects

^2 ^P-values generated for each group by ANOVA with treatment group as factor.

### Weight Maintenance and Baseline Values

Subjects maintained their weight during the trial with no significant weight loss and no significant weight differences between treatment groups. At baseline subjects mean body weight was 90.5, 91.0 and 90.8 kg with a 12 week mean change of 0.7, -0.3 and 0.2 kg for the 3 g/d, 6 g/d and placebo groups respectively (p = 0.475). No significant differences were found between the groups for baseline fasting glucose, insulin, or HOMA-IR levels (Table 4).

**Table 4:** Fasting Glucose, Insulin and Homeostatic Model Assessment of Insulin Resistance (HOMA-IR)^1^

Parameter	Visit	Placebo N = 14	3 g/d BBG N = 15	6 g/d BBG N = 15	p-value2	Pairwise Comparisons3
**Fasting Plasma Glucose (mg/dL)**	**Week 0**	102.4 (2.4)	103.7 (3.9)	99.1 (2.3)	0.775 (r)	3 g/d: 0.977; 6 g/d: 0.833
	**Week 12**	110.1 (4.4)	112.0 (7.6)	100.6 (3.1)		
	**Change**	7.6 (4.7)	8.3 (7.3)	1.5 (3.0)	0.399 (r)	3 g/d: 0.577; 6 g/d: 0.305
**Fasting Insulin (uIU/mL)**	**Week 0**	6.5 (1.1)	8.7 (1.6)	8.3 (1.6)	0.752 (r)	3 g/d: 0.671; 6 g/d: 0.856
	**Week 12**	8.6 (1.8)	8.3(1.6)	6.7 (1.3)		
	**Change**	2.1 (1.2)	-0.4 (.8)	-1.6 (1.0)	0.014 (r)	3 g/d: 0.091; 6 g/d: 0.008
**HOMA-IR**	**Week 0**	1.7 (0.3)	2.3 (0.5)	2.1 (0.4)	0.834 (r)	3 g/d: 0.771; 6 g/d: 0.952
	**Week 12**	2.4 (0.5)	2.3 (0.5)	1.7 (0.3)		
	**Change**	0.7 (0.3)	-0.0 (0.3)	-0.4 (0.3)	0.025 (r)	3 g/d: 0.130; 6 g/d: 0.014
**Matsuda Index**	**Week 0**	16.0 (2.6)	18.1 (4.1)	16.7 (3.8)		
	**Week 12**	14.1 (3.4)	17.4 (4.7)	17.5 (3.7)		
	**%** **Change**	-13.4 (9.4)	-3.3 (10.9)	18.1 (12.2)	0.928 (r)	3 g/d: 0.901; 6 g/d: 0.949

Values presented are Mean (Standard Error of Mean); Change = Change from Week 0 to 12

^1 ^Data represented includes only subjects with end of study values

^2^P-values generated from final analysis of variance model; (r) indicates values were ranked prior to analysis.

^3^Pairwise comparisons to Placebo derived by Dunnett's procedure

### Fasting Plasma Glucose

Mean baseline fasting plasma glucose values were similar in the three groups with values ranging between 99.1 mg/dl to 103.7 mg/dl (Table 4). Although not statistically significant, over the 12 weeks of the trial, the mean fasting glucose levels increased in all groups, but numerically less so in the 6 g/d BBG group (7.6, 8.3 and 1.5 mg/dl for the placebo, 3 g/d BBG and 6 g/d BBG groups respectively).

### Fasting Plasma Insulin, HOMA-IR and Matsuda Index

Baseline fasting plasma insulin values were similar in the three groups with mean values ranging between 6.5 and 8.7 uIU/ml (Table 4). The group receiving 6 g/d BBG for 12 weeks had significantly reduced fasting serum insulin values by a mean of 7.8%; decreasing from a mean of 8.3 to 6.7 uIU/mL compared to placebo which increased from a mean of 6.5 to 8.6 uIU/mL (p = 0.008). Similarly, 6 g/d of BBG significantly reduced HOMA-IR from week 0 to week 12 from a mean of 2.1 to a mean of 1.7 compared to placebo which increased from a mean of 1.7 to a mean of 2.4 (p = 0.014). The Matsuda Index, which is inversely related to the HOMA-IR, [24,25] showed a mean decrease of 3.3% for the 3 g/d and a mean increase of 18.1% for the 6 g/d BBG compared to the placebo which decreased a mean of 13.4% (p = 0.129).

### Post- OGTT Plasma Glucose and Insulin

**Figure 2:**
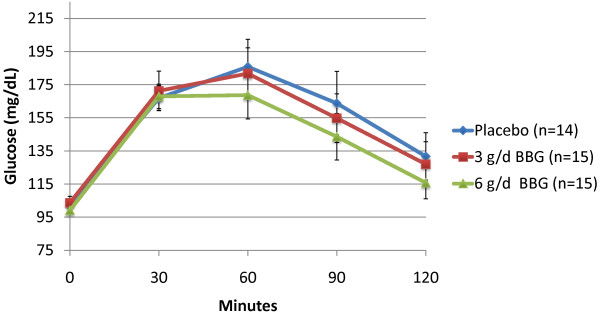
**Post Oral Glucose Tolerance Testing (OGTT) Plasma Glucose: Baseline (Mean +/- SEM)**.

**Figure 3:**
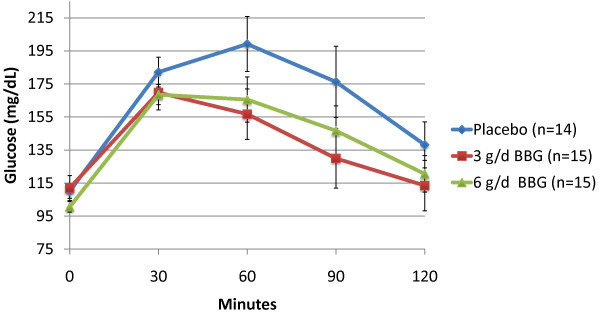
**Post Oral Glucose Tolerance Testing (OGTT) Plasma Glucose: Week 12 (Mean +/- SEM)**.

**Figure 4:**
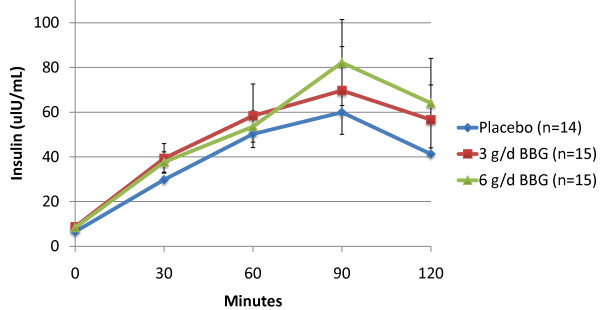
**Post Oral Glucose Tolerance Testing (OGTT) Insulin: Baseline (Mean +/- SEM)**.

**Figure 5:**
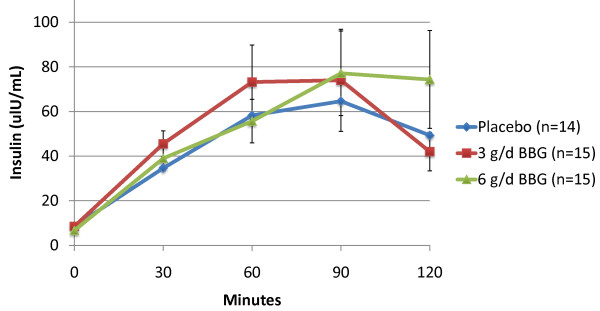
**Post Oral Glucose Tolerance Testing (OGTT) Insulin: Week 12 (Mean +/- SEM)**.

Subjects receiving 3 g/d BBG for 12 weeks experienced a mean 10.2% reduction in glucose iAUC during the OGTT (-1876 min* mg/dl) compared to subjects receiving placebo who experienced a mean 7.5% increase (1234 min* mg/dl) (p = 0.011). The 6 g/d BBG group showed a similar trend (mean = -76 min* mg/dl), but was not statistically significant (p = 0.373) (Figures 2 and 3). Mean insulin iAUC changes from week 0 to week 12 were 134, 739 and -59 min* uIU/mL for the placebo, 3 g/d and 6 g/d respectively (not significant) (Figures 4 and 5).

### Adverse Events

A total of 69 adverse events were reported including 17 in the placebo group (7 subjects), 18 with 3 g/s BBG (7 subjects) and 34 with 6 g/d BBG (10 subjects) (p = 0.540). The most common adverse events included diarrhea, abdominal distension and flatulence. These adverse events were typically mild and self-limited, with no significant differences between the study groups. No reported serious adverse events occurred during this trial and no subjects withdrew from this study due to an adverse event.

## Discussion

In this study, 6 g/d of reduced viscosity BBG consumption over 12 weeks improved insulin sensitivity (i.e. reduced fasting insulin and HOMA-IR) in generally healthy study subjects with baseline hyperglycemia, but without a prior diagnosis of diabetes mellitus. The fasting plasma glucose levels had less of a numeric rise among the group receiving 12 weeks of 6 g/d BBG compared to the placebo group (not significant). Because this trial was designed to ensure study subjects underwent protocol-directed maintenance of body weight, it is noteworthy that the metabolic findings occurred without significant weight changes in any of the study groups.

Acute, single-meal studies or observations for less that 24-hours, have documented that acute ingestion of BBG is associated with significant decreases in postprandial glycemia and insulinemia [16-20,26-37]. Four short-term clinical trials of 4 to 6 week duration have not documented changes in fasting blood glucose and insulin values comparing BBG consumption to control foods or products [38-41]. Generally, these studies were of suboptimal duration and most were not powered to detect the small changes that might be expected in non-diabetic subjects; however, one study [38] found a significant reduction in postprandial blood glucose iAUC values after four weeks of treatment in the six subjects in that cross over study. One, longer-term, cross over study with a 12-week treatment duration found postprandial insulin values were significantly increased while the favorable reductions in fasting plasma glucose (-7.3%), fasting plasma insulin (-5.9%) and post-prandial glucose values (-20%) were not statistically significant for the eleven T2DM male subjects in this trial [42].

Consistent with prior studies [10-20,26-38,42], this study showed similar improvements in some but not all measured parameters of glucose homeostasis. Of interest, prior trials inconsistently suggested dietary fiber consumption may increase satiety and decrease caloric intake [32-36]. If a reduction in body weight were allowed through utilizing a different study design than this study, and if weight loss were achieved, then it might be argued that any improvement in measures of glucose metabolism were simply due to a reduction in body weight. However, since this study demonstrated BBG improved glycemic parameters despite no change in body weight, this suggests BBG in humans may have glycemic benefits beyond weight loss, as found with soluble fibers studied in animals [43,44].

Although the data are not always consistent, the degree by which soluble fibers favorably affect metabolic parameters (fasting glucose or insulin, postprandial glucose or insulin) may be somewhat dependent upon the viscosity [40-42]. Like other viscous soluble fibers, BBG ingestion reduces postprandial glycemia and insulinemia [15]. Possible mechanisms describing how viscous soluble fibers may improve glucose metabolism and insulin sensitivity include slow absorption of glucose in the small intestine, colonic fermentation, and potential effects upon gastrointestinal hormones [9]. Colon fermentation after consuming a meal containing indigestible carbohydrates (like barley beta-glucan) may contribute to subsequent meal improvements in postprandial glycemia [45]. Increased serum short chain fatty acid concentrations resulting from colonic fermentation of soluble fibers may have favorable effects on hepatic lipid metabolism and improve glucose metabolism [46,47].

Limitations of this study relate to the relatively small sample size. The findings support potential confirmatory trials with this same product formulation and related formulations of this product. Another limitation is that while this trial was longer than many previous trials with this agent, even longer trials may provide more information regarding the clinical implications regarding the onset of type 2 diabetes mellitus. Future studies might evaluate both longer terms of treatment (perhaps a year or more in combination with weight loss programs for those who are overweight and balanced diet counseling for all) as well as additional blood sampling times after treatment since the pharmacodynamic effects of BBG may last longer than the initial 2 hours sampled in this trial.

From a safety and tolerability perspective, the flavored water beverage containing BBG was generally well tolerated, with no serious adverse experiences and no significant differences between groups for adverse events.

## Conclusions

In summary, this study supports that the 6 g/d BBG beverage consumed over 12 weeks improves insulin sensitivity among hyperglycemic individuals who have no prior diagnosis of diabetes mellitus and no change in body weight. This study suggests BBG may slow the deterioration of insulin sensitivity for individuals at increased risk for diabetes mellitus.

## List of abbreviations

(AE): Adverse Event; (ANOVA): Analysis of Variance; (BBG): Barley β-Glucan; (BMI): Body Mass Index; (BP): Blood Pressure; (bpm): Beats Per Minute; (d): day; (dl): Deciliter; (GRAS): Generally Regarded as Safe; (g): gram; (HbA1c): Hemoglobin A1c; (HOMA-IR): homeostasis model assessment of insulin resistance; (Ht): Height; (HR): Heart Rate; (iAUC): incremental Area Under the Curve; (kg): Kilogram; (m): Meter, (mg): Milligram; (ml): Milliliter; (OGTT): Oral Glucose Tolerance Test; (SAE): Serious Adverse Event; (SEM): Standard Error of the Mean; (TLC): Therapeutic Lifestyle Changes; (T2DM): Type 2 Diabetes Mellitus; (wt): Weight.

## Competing interests

HB: research site received research grants for clinical trial conduct, JLF: business received research grants for protocol design, project management, data analysis and reporting for this clinical trial, MB: business received research grants for statistical planning and analysis, CW: research site received research grants for clinical trial conduct, LK: employee of Cargill. WS: employee of Cargill, and JWA: received payment as a Consultant Medical Monitor and for protocol development for this clinical trial.

## Authors' contributions

JLF, MB, LK and JWA designed the clinical trial protocol; HB, JLF, MB, LK, and JWA conducted the research and interpreted the data; CW and WS also conducted research; JLF and MB analyzed data; JLF drafted manuscript; HB, JLF, MB, CW, LK, WS and JWA contributed revisions to the manuscript; JLF had primary responsibility for the final content of the manuscript; and all authors read and approved the final manuscript and decided to submit the manuscript for publication.
